# Navigated iliac screw placement may reduce radiation and OR time in lumbopelvic fixation of unstable complex sacral fractures

**DOI:** 10.1007/s00590-021-02892-7

**Published:** 2021-02-16

**Authors:** M. F. Hoffmann, E. Yilmaz, D. C. Norvel, T. A. Schildhauer

**Affiliations:** 1 Berufsgenossenschaftliches Universitätsklinikum Bergmannsheil Bochum, Bürkle-de-la-Camp-Platz 1, 44789 Bochum, Germany; 2 Spectrum Research, Inc., Tacoma, WA USA

**Keywords:** Navigation, Radiation, Lumbopelvic fixation, Fluoroscopy

## Abstract

**Purpose:**

Instability of the posterior pelvic ring may be stabilized by lumbopelvic fixation. The optimal osseous corridor for iliac screw placement from the posterior superior iliac spine to the anterior inferior iliac spine requires multiple ap- and lateral-views with additional obturator-outlet and -inlet views.

The purpose of this study was to determine if navigated iliac screw placement for lumbopelvic fixation influences surgical time, fluoroscopy time, radiation exposure, and complication rates.

**Methods:**

Bilateral lumbopelvic fixation was performed in 63 patients. Implants were inserted as previously described by Schildhauer. A passive optoelectronic navigation system with surface matching on L4 was utilized for navigated iliac screw placement. To compare groups, demographics were assessed. Operative time, fluoroscopic time, and radiation were delineated.

**Results:**

Conventional fluoroscopic imaging for lumbopelvic fixation was performed in 32 patients and 31 patients underwent the procedure with navigated iliac screw placement. No differences were found between the groups regarding demographics, comorbidities, or additional surgical procedures.

Utilization of navigation led to fluoroscopy time reduction of more than 50% (3.2 vs. 8.6 min.; *p* < 0.001) resulting in reduced radiation (2004.5 vs. 5130.8 Gy*cm^2^; *p* < 0.001). Operative time was reduced in the navigation group (176.7 vs. 227.4 min; *p* = 0.002) despite the necessity of additional surface referencing.

**Conclusion:**

For iliac screws, identifying the correct entry point and angle of implantation requires detailed anatomic knowledge and multiple radiographic views. In our study, additional navigation reduced operative time and fluoroscopy time resulting in a significant reduction of radiation exposure for patients and OR personnel.

## Introduction

**Fig. 1:**
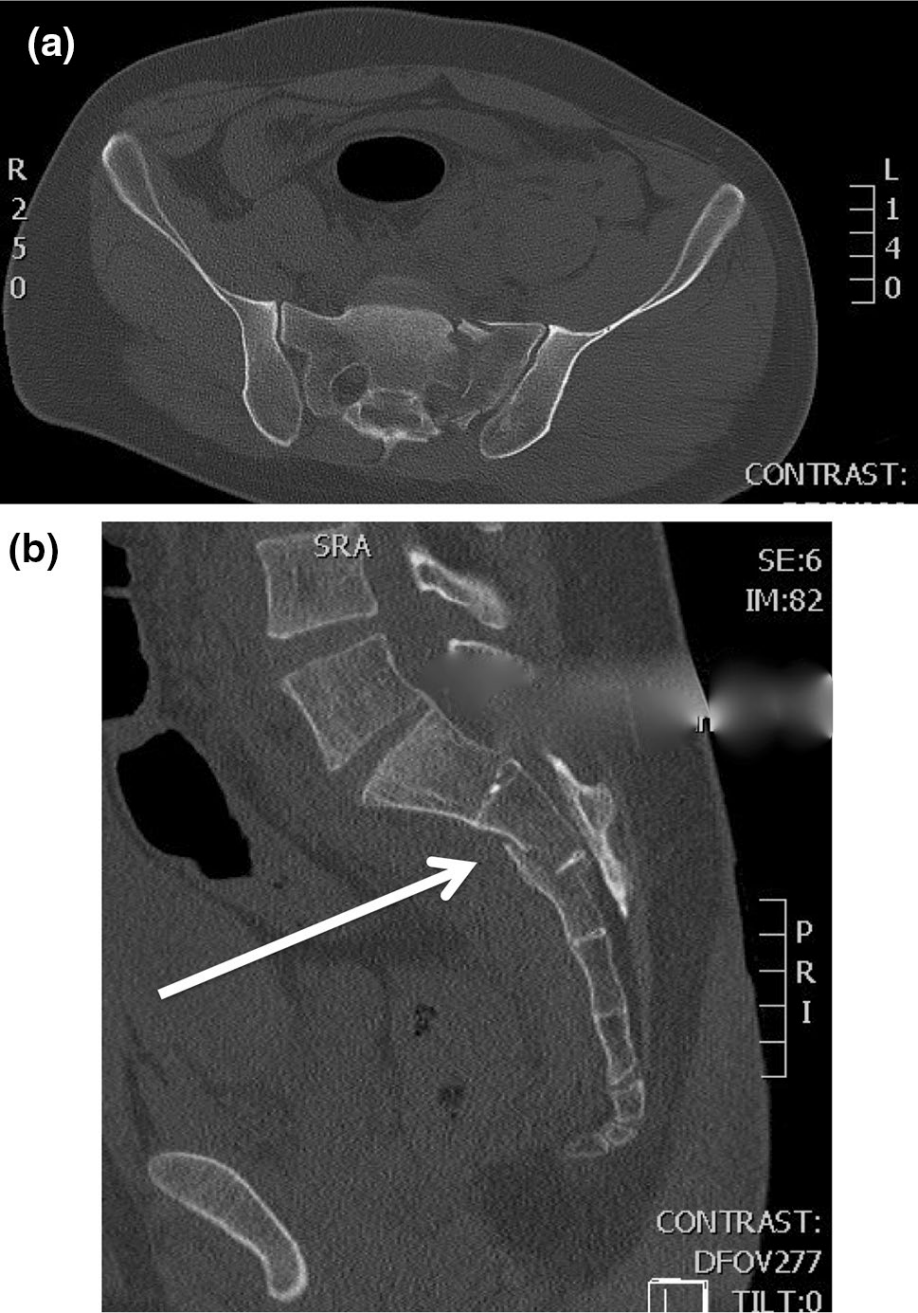
CT scans of all patients have been performed. **a** Demonstrates a comminuted sacral fracture. The sagittal reconstruction (**b**) shows the horizontal fracture line leading to rotational instability

In trauma patients, the sacrum is the keystone of the pelvic ring [1]. Despite multiple operative stabilization techniques including open or percutaneous iliosacral screw osteosynthesis, tension band transiliac plate osteosynthesis, transiliac bars, and local plate osteosynthesis, fixation of sacral fractures continues to be challenging due to complex local anatomy, unique biomechanical forces, and often poor bone quality [2, 3]. Especially in severe comminuted sacral fractures and fractures with horizontal fracture lines (Fig. 1a, b) lumbopelvic fixation introduced by Trenz et al. [4] provides superior stability [5] and allows immediate weight-bearing while unloading the area of injury [6–8]. Pedicle screw placement is a common procedure and presents no relevant difficulties. Identifying the correct entry point for the iliac screws and the correct angle of implantation in all planes is crucial, requires detailed anatomic knowledge, and multiple radiographic views [9]. The optimal osseous corridor for iliac screw placement requires multiple posteroanterior (PA) and lateral views (LAT) with additional obturator outlet and obturator inlet views. Obtaining the correct views results in increased fluoroscopy time and radiation exposure for the patient and OR personnel.

Different navigation systems based on fluoroscopy or CT scans are established in orthopedic surgery and routinely utilized for pedicle screw placement. Previous studies suggest that computer assisted and navigated surgery for screw placement in pelvic fractures is advantageous and may result in increased accuracy of screw placement, reduction of fluoroscopy time, OR time, and radiation exposure [8, 10–12].

Therefore, the purpose of this study was to determine if navigated iliac screw placement for lumbopelvic fixation influences (1) surgical time, (2) fluoroscopy time, (3) radiation exposure, (4) accuracy of screw placement, and (5) complication rates.

## Materials and methods

**Fig. 2:**
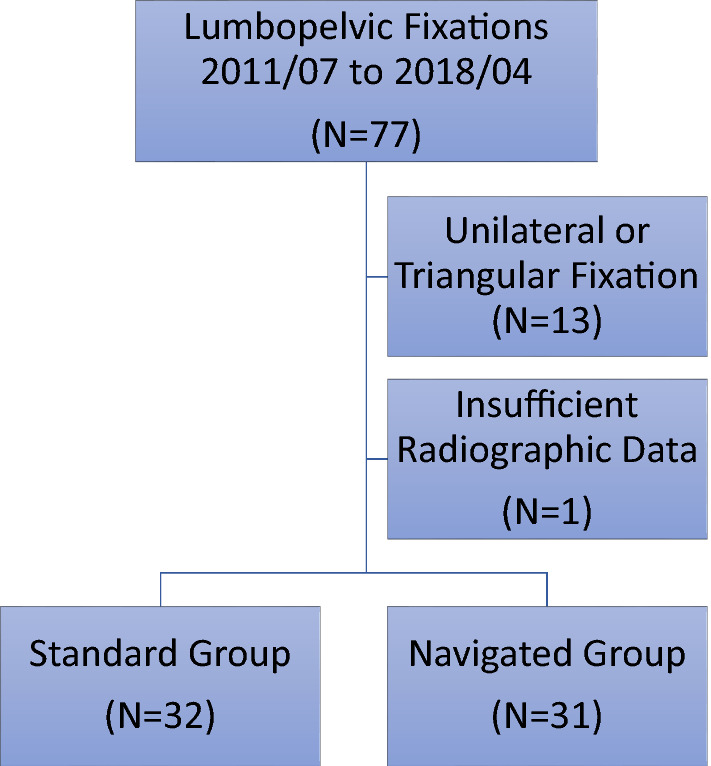
Diagram illustrating the excluded patients and study groups

This study was an Institutional Review Board approved retrospective cohort study of consecutive patients undergoing surgical treatment of unstable sacral fractures treated with lumbopelvic fixation in a single level I trauma center. During the study period, we identified 77 patients that had lumbopelvic fixation for pelvic fractures or instabilities from July 2011 through April 2018. Inclusion criteria were: bilateral surgical treatment for unstable sacral fractures or sacral instability and age equal to or older than 18 years. Exclusion criteria were insufficient medical and/or radiographic data, or triangular fixation (Fig. 2).

Each patient had three preoperative views of the pelvis. These were an anteroposterior (AP) view with the patient supine, a pelvic inlet view, and an outlet view. Inlet and outlet views were performed for assessing rotational, translational, and vertical displacement. Each patient had a CT scan with reconstruction of the injured pelvis that provided information on both extent of the injury and the magnitude of the displacement of the sacroiliac joint, the sacrum, or the iliac wing. Furthermore, the CT scan was utilized in the navigated group as the dataset for the 3D navigation and reconstruction.

Patients were positioned prone on a radiolucent table with appropriate eye protection and sequential compression devices. The entire posterior aspect of the pelvis and gluteal area was prepped and draped. A midline incision was performed down to the posterior elements. Attention to detail was maintained to avoid dural or neural element injury through the fracture sites. The operative approaches to the pelvis were tailored to each patient based on the particular pattern of the injury, location of the fracture, associated injuries, and soft tissue involvement [13]. Lumbopelvic implants (USS II, DePuySynthes, Paoli, PA) were inserted as described by Schildhauer through a midline incision [14]. The iliac screws were positioned in the bony canal between the posterior superior iliac spine (PSIS) and the anterior inferior iliac spine (AIIS), connected to pedicle screws by vertical rods and additionally stabilized by a transverse rod to prevent vertical shear.

Patients were either operated in standard fashion utilizing intraoperative standard fluoroscopic views or underwent computer assisted and navigated surgery for iliac screw placement by the first author.

**Fig. 3:**
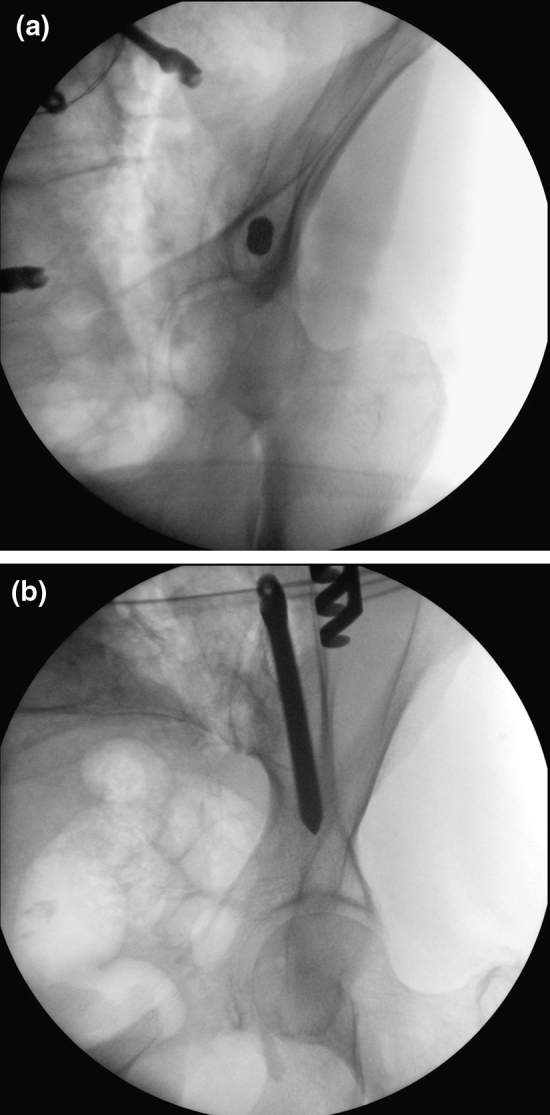
**a**, **b** The intraoperative fluoroscopic views demonstrating the intraosseous placement of the iliac screws

**Fig. 4:**
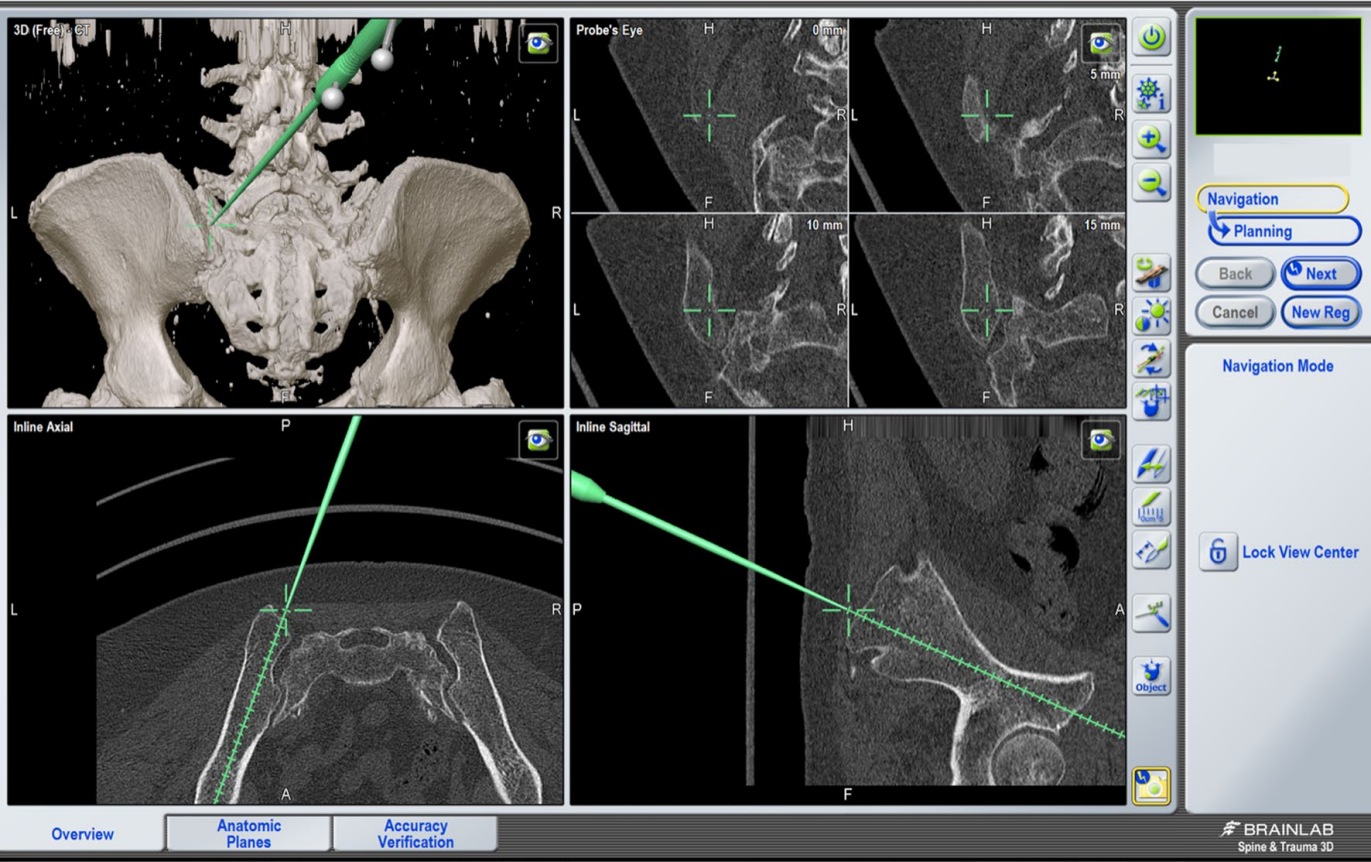
Intraoperative view of the 3D navigation for iliac screw placement

Standard iliac screw placement was performed in 32 patients utilizing anteroposterior (AP) and lateral views (LAT) with additional obturator outlet and obturator inlet views [9] (Fig. 3a, b). In 31 patients, a passive optoelectronic navigation system (Curve, Brainlab, Germany) was used for iliac screw placement (Fig. 4). Surface registration was performed on the lamina and spinous process of L4 for the matching procedure. Iliac screws were placed before any reduction maneuver. In both groups, no open reduction of the sacrum was required.

In all patients, a recession was created for the screw head, end cap, and connector rod in an attempt to reduce screw head prominence. Screw size was based upon length from the recessed entrance of the PSIS, along the sciatic buttress, and ending at the anterior inferior iliac spine (AIIS) and was 7.0 or 8.0 mm × 110–130 mm (USS II, Titanium, DePuySynthes, Paoli, PA). Neural decompression was performed based on the fracture pattern or neurologic symptoms. Patients with ligamentous injury involving the L5-S1 interval or had extensive posterior neural decompression involving the S1 facets and lamina underwent an associated L5-S1 posterolateral arthrodesis. Approaches were closed over drains and in anatomical layers. Skin was closed with vertical Allgower-Donati 3.0 nylon (Ethibond, Johnson & Johnson, Norderstedt, Germany) sutures.

To compare groups, demographics were assessed, operative time, fluoroscopic time, radiation, and complications were delineated. Screw misplacement was assumed when screws were revised. Deep infection was defined as an infection requiring operative excisional debridement and antibiotic administration.

Data were analyzed using PASW® 18 (IBM, Armonk, NY). Descriptive statistics were completed. Chi-square and *t* tests were used to compare those that had navigation aid versus those that did not, based on demographic data, potential contributing factors, OR time, fluoroscopy time, and radiation.

## Results

Sixty-three (63) patients were included with a mean age of 58 years (range 18–87). There were 27/63 (43%) males and 36/63 (57%) females with a BMI of 26.2 ± 5.6 kg/m^2^. Length of hospital stay was averaged 33 ± 26.2 days. Reasons for instabilities and surgeries are listed in Table 1. Using the OTA/AO classification, all fractures were classified as 61 type C fractures.

**Table 1:** Lumbopelvic fixation was performed for different instability reasons

Reason for surgery	Standard group	Navigation group	Significance
Nonunion or insufficient previous fixation	5 (16%)	9 (29%)	*p* = 0.12
Spondylodiscitis	7 (22%)	2 (6%)	*p* = 0.08
Tumor	1 (3%)	0 (0%)	*p* = 0.32
Fracture	19 (59%)	20 (65%)	*p* = 0.58

### Groups

During the study period, 32 patients underwent bilateral lumbopelvic fixation utilizing conventional fluoroscopic imaging alone (*standard group*) and 31 patients underwent the procedure with 3D navigated iliac screw placement (*navigation group*). No differences were found between the two groups regarding age (59.9 ± 18.4 vs. 56.6 ± 18.7 years; *p* = 0.49), BMI (26.0 ± 6.0 vs. 26.3 ± 5.1 kg/m2; *p* = 0.81), gender (59% vs. 58% females; *p* = 0.59), length of hospital stay (35.2 ± 24.7 vs. 30.5 ± 27.9 days; *p* = 0.49), or comorbidities (Table 2).

**Table 2:** Comparison of demographics between standard and navigations groups

	Standard group	Navigation group	Significance
Age (years)	59.9 (SD18.4)	56.6 (SD 18.7)	*p* = 0.49
BMI (kg/m^2^)	26.0 (SD 6.0)	26.3 (SD 5.1)	*p* = 0.81
Females (%)	59	58	*p* = 0.59
Length of hospital stay (days)	35.2 (SD 24.7)	30.5 (SD 27.9)	*p* = 0.49
Osteoporosis (%)	25	39	*p* = 0.24
Cardiovascular disease (%)	53	39	*p* = 0.26
Diabetes (%)	9	13	*p* = 0.66

*SD* =Standard Deviation

Comparing screw length and diameter of the iliac screws, screw length averaged 111.6 ± 7.7 mm in the standard group compared to 108.4 ± 8.2 mm in the navigated group with a median of 110 mm in both groups (*p* = 0.12). Screw diameter averaged 7.9 ± 0.47 mm with a median of 8 mm in the standard group. All navigated screws were 8 mm in diameter (*p* = 0.40). Regarding additional procedures, in the standard group 15 patients (47%) underwent additional decompression compared to 12 patients (39%) in the navigated group (*p* = 0.51). The number of inserted pedicle screws averaged 2.92 in the standard group (median 2) with 66% at the L4 and/or L5 level and 3.0 in the navigated group (median 2) (*p* = 0.88).

### Surgical time

**Fig. 5:**
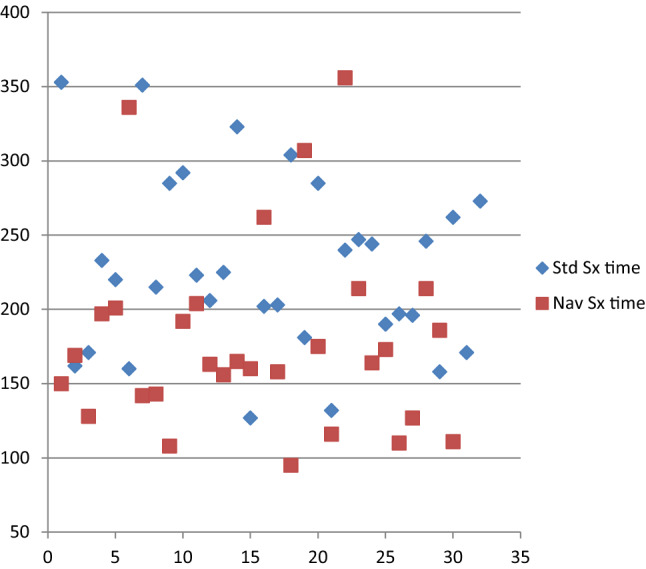
Distribution of surgery time (minutes) for standard and navigated fixation

Surgical time varied from 95 to 356 min with an average of 203 min. Comparing both groups, OR time averaged 227.4 ± 59.0 min in the standard group. In the navigated group, OR times was 176.7 ± 64.9 min (*p* = 0.002) (Fig. 5).

### Fluoroscopy time

Fluoroscopy time was 6.02 ± 5.98 min for all patients combined. During standard lumbopelvic fixation an average of 8.6 ± 7.2 min of fluoroscopy was utilized compared to 3.2 ± 2.0 min in navigated cases (*p* < 0.001).

### Radiation exposure

**Fig. 6:**
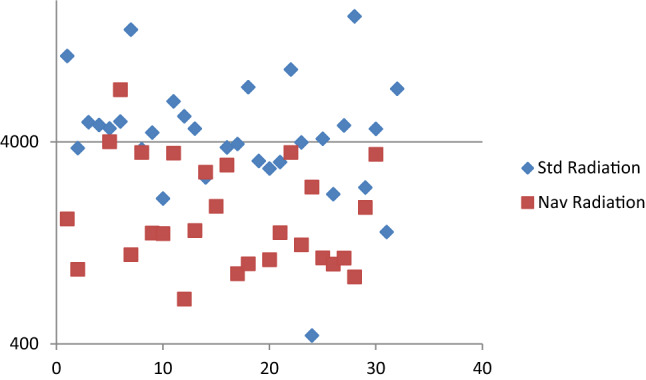
Distribution of radiation (Gy*cm^2^) for standard and navigated fixation

Patients and OR personnel were exposed to 3696.77 ± 3142.86 Gy*cm^2^ during lumbopelvic fixations. Average radiation exposure during surgery was reduced by navigation from 5130.8 ± 3496 Gy*cm^2^ to 2004.5 ± 1465.2 Gy*cm^2^ (*p* < 0.001) (Fig. 6).

### Complications and accuracy of screw placement

Additional surgeries were required in twenty-six patients (41.3%). The reason for revision surgery was irrigation and debridement for hematoma or infection in 15 cases (23.8%). Comparing both groups, additional surgeries were performed in 17 patients (53%) in the standard group and nine patients (30%) in the navigated group (*p* = 0.07). Infection was reduced in the navigated group compared to the standard group (7% vs. 28%, respectively; *p* = 0.03). The incidence of infection was not related to the OR time (201 min vs. 204 min). One of the 63 patients had a malpositioned pedicle screw that needed revision (1.6%). The malpositioned screw happened to be in the standard group (3% vs. 0%, respectively) but that was not significant (*p* = 0.33). Other reasons for revision surgeries were additional bone grafting for delayed spondylodesis (4), a disconnected drainage (1), extended decompression for persistent neurologic symptoms (1), and three patients required hardware exchange for broken rods in nonunion formation. Surprisingly, the iliac screws were still well-fixed and were not replaced during revision surgery. No iatrogenic neural injuries were recorded. One patient returned for hardware removal 1 year after the initial surgery.

## Discussion

The key requirement of successful posterior pelvic ring repair is a proper alignment among the ilium, sacrum, and lumbar spine [15] that is stable enough to counterbalance translational and rotational forces in vertical and horizontal directions [6]. Lumbopelvic fixation transfers vertical loads from the ilium directly to the lumbar spine and prevents flexion of the pelvis [16]. The surgical technique is demanding with numerous potential complications [17]. In appropriately selected patients, high success rates can be achieved when it is performed systematically [2, 18]. Biomechanical studies have confirmed that segmental lumbopelvic stabilization provides stable fixation of the posterior pelvic ring while unloading the area of injury [6, 19]. Pedicle screw placement usually poses no difficulty and is performed routinely in most orthopedic clinics. One of the keystones for success of the lumbopelvic technique is the insertion of the iliac screws. Reliable screw positioning can be achieved utilizing multiple fluoroscopic views [9, 14]. In the current study, only one malpositioned screw was observed in the standard group but that was no significant difference compared to the navigated group.

Standard screw placement may result in long radiation exposure, which is already increased for spinal surgeons and orthopedic OR staff [20–22]. Previous studies did show that navigation offers the possibility to reduce radiation exposure [23]. The current study shows that fluoroscopy time could be reduced by more than 60% from an average of 8.6 ± 7.2 min during standard lumbopelvic fixation to 3.2 ± 2.0 min in procedures with navigated screw placement. This resulted in a radiation reduction of more than 50% by reducing the average radiation exposure during surgery by navigation from 5131 Gy*cm^2^ to 2005 Gy*cm^2^. In addition, the utilization of the surface matching combined with 3D navigation did not require additional CT scans. The necessary data were retrieved from the routinely performed CT scan that provided preoperative information on the extent of the injury.

Increased muscle mobilization and potential devitalization may increase the risk for deep hematoma formation and wound healing problems [17, 24]. In previous studies, 26% of the patients required early secondary surgical procedures for wound-related complications [7]. In the current study, soft tissue-related complications were encountered in 24%. Utilizing a more submuscular or minimal invasive approach to the PSIS may reduce soft tissue complications [25]. In our study, navigation did result in a reduced infection rate in the navigated group (7%) compared to the standard group (28%) (*p* = 0.03). The reason remains unclear and no differences were documented in our medical charts. Localizing the entry point for the iliac screw with the pointer of the navigation system and a direct more submuscular approach may reduce soft tissue damage. Further studies determining factors that reduce wound healing problems are warranted.

We must admit limitations of our study. The major limitation of this study was its retrospective design. Therefore, no power analysis was conducted previously to assess sample sizes. This patient series represents complex instabilities all undergoing lumbopelvic fixation. All instabilities were complex and problematic being not amenable to traditional posterior pelvic ring fixation options. As a result, we also must acknowledge that a reasonable comparison of radiographic or clinical outcomes in a variety of instabilities was not possible.

The strength of this study is the number of patients included. To our knowledge, this study is the largest series comparing standard screw placement with navigated screw positioning in lumbopelvic fixations. All procedures were performed by trauma fellowship-trained orthopedic surgeons who have consistent treatment philosophies in one high volume level I trauma center. Navigated procedures were performed by the first author while the remaining surgeons continued standard screw placement. The initiation of the navigated procedure and the learning curve is included in the study. Therefore, this study has the potential to reflect realistic differences in OR time and fluoroscopy.

## Conclusion

Fixation of sacral fractures continues to be challenging due to complex local anatomy. Especially in severe comminuted sacral fractures lumbopelvic fixation provides superior stability and allows immediate weight-bearing. For iliac screws, identifying the correct entry point and angle of implantation in all planes requires detailed anatomic knowledge and multiple radiographic views. For lumbopelvic fixation, 3D navigation may help to reduce operative time and fluoroscopy time resulting in a significant reduction of radiation exposure for the patient and OR personnel following the principle to minimize occupational radiation exposure “as low as reasonably achievable” [22].

## Data Availability

The datasets used and/or analyzed during the current study are available from the corresponding author on reasonable request.
